# Bioactive Molecules from Extreme Environments

**DOI:** 10.3390/md18120640

**Published:** 2020-12-14

**Authors:** Daniela Giordano

**Affiliations:** 1 Institute of Biosciences and BioResources (IBBR), CNR, Via Pietro Castellino 111, 80131 Napoli, Italy; daniela.giordano@ibbr.cnr.it; 2 Department of Marine Biotechnology, Stazione Zoologica Anton Dohrn (SZN), Villa Comunale, 80121 Napoli, Italy

**Keywords:** Arctic/Antarctic, deep-sea, deep hypersaline anoxic basin, cold-adapted bacteria, halophilic microorganisms, marine natural product, enzyme, carotenoid, silver nanoparticle, marine bioprospecting

## Abstract

Marine organisms inhabiting extreme habitats are a promising reservoir of bioactive compounds for drug discovery. Extreme environments, i.e., polar and hot regions, deep sea, hydrothermal vents, marine areas of high pressure or high salinity, experience conditions close to the limit of life. In these marine ecosystems, “hot spots” of biodiversity, organisms have adopted a huge variety of strategies to cope with such harsh conditions, such as the production of bioactive molecules potentially valuable for biotechnological applications and for pharmaceutical, nutraceutical and cosmeceutical sectors. Many enzymes isolated from extreme environments may be of great interest in the detergent, textile, paper and food industries. Marine natural products produced by organisms evolved under hostile conditions exhibit a wide structural diversity and biological activities. In fact, they exert antimicrobial, anticancer, antioxidant and anti-inflammatory activities. The aim of this Special Issue “Bioactive Molecules from Extreme Environments” was to provide the most recent findings on bioactive molecules as well as enzymes isolated from extreme environments, to be used in biotechnological discovery pipelines and pharmaceutical applications, in an effort to encourage further research in these extreme habitats.

## 1. Foreword

Marine organisms produce a huge variety of natural products that confer important advantages, either as antibiotics or by allowing communication with other organisms and with the environment. These molecules have long been exploited in medical fields as antioxidants, antimicrobial and anticancer agents, and in biotechnological applications, attracting an increasing commercial interest from the biotech sector [1]. In 2016, 1277 new marine compounds were reported exerting interesting biological activities, and many of these are of bacterial origin [2]. The global market for marine-derived drugs is expected to be USD 2745.80 million by 2025 [3]. However, to date, few molecules isolated from marine organisms have been approved [4] and many marine ecosystems remain unexplored. 

Due to the limited accessibility and remoteness, extreme marine environments are still largely underexploited in comparison with terrestrial ecosystems. Recent advances in the sampling and accessibility of extreme areas, together with the development of omics technologies, have opened new avenues for drug discovery. These remote habitats are promising reservoirs of biotechnological and biomedical applicability, and represent a good opportunity for compound discovery and bioprospecting. Marine organisms inhabiting extreme environments have adopted unique survival strategies for growing and reproducing under hostile conditions, biosynthesizing an array of biomolecules potentially valuable for many applications in the biotechnological sector, in the pharmaceutical and cosmeceutical industries and in the bioremediation. 

Extreme environments are exposed to one or more environmental parameters, i.e., temperature, salinity, osmolarity, UV radiation, pressure or pH, showing values close to the limit of life. Polar and hot regions, deep sea, hydrothermal vents, marine areas of high pressure or high salinity, and acidic and alkaline regions are examples of extreme environments. In the Arctic and Antarctic regions, the sea ice can reach up to ~13% of the Earth’s total surface. The Arctic Ocean is mainly characterized by light seasonality and cold temperatures with winter extremes, whereas Antarctica is considered as the driest, windiest and coldest place on Earth, completely isolated, geographically and thermally, from the other continents. Polar marine life has adapted to thrive in the ocean’s most inhospitable conditions, where extremes of pressure and temperature and the absence of light have selected species with a unique range of bioactive compounds, either enzymes [5] or secondary metabolites [6]. 

The deep sea, below a depth of 1000 m, is recognized as an extreme environment. It is characterized by the absence of sunlight and the presence of low temperatures and high hydrostatic pressures. This environment becomes even more extreme in particular conditions, with extremely high temperatures of >400 °C in deep-sea hydrothermal vents, and with extremely high salinities and high pressures in deep hypersaline anoxic basins (DHABs). These habitats have been discovered on the sea floor in different oceanic regions, such as the Red Sea, the eastern Mediterranean Sea and the Gulf of Mexico. New DHABs, the Thetis, Kyros and Haephestus basins in the Mediterranean Sea, have been recently discovered. DHABs are microbial “hotspot”, with dense microbial populations of unique bacterial lineages more metabolically active than those of the adjacent layers [7]. Deep-sea extremophiles, able to proliferate under these challenging parameters (pressure and temperature, pH, salinity and redox potential), have adopted a variety of strategies to cope with these extreme environments, such as the production of extremozymes, with thermal or cold adaptability, salt tolerance and/or pressure tolerance, and secondary metabolites with biomedical applications.

The majority of marine bioactive compounds comes from microorganisms as a prolific resource for novel chemistry and the sustainable production of bioactive compounds, bypassing the problem of the recollection of samples from the field, which is required for macroorganisms. Recently, the increasing number of whole microbial genomes and the development of genome mining approaches have accelerated the discovery of novel bioactive molecules, overcoming the requirement for the isolation or cultivation of microorganisms [8]. 

## 2. The Special Issue

The Special Issue “Bioactive Molecules from Extreme Environments” was aimed at collecting papers regarding bioactive molecules and enzymes isolated from organisms inhabiting extreme environments being used in biotechnological discovery pipelines and pharmaceutical applications. Special attention was paid to the species biodiversity of extreme habitats as a promising reservoir of untapped compounds with biotechnological potential. 

In total, eleven articles were accepted and included in the Special Issue. Most of the articles in this Special Issue were focused on marine microorganisms, bacteria and fungi, with their chemical diversity and active metabolites able to cope with harsh habitats. 

Five articles in this Special Issue were focused on enzymes from microorganisms in extreme environments. Many enzymes have been evaluated for industrial applications, for the production of pharmaceuticals, foods, beverages, paper, as well as in textile and leather processing and waste-water treatment. However, only a few of these are able to meet the industrial demands that include tolerance of harsh conditions of temperature, pH, salinity and pressure, maintaining high conversion rate and reproducibility. The market for enzymes is expected to reach USD 7.0 billion by 2023, from USD 5.5 billion in 2018 [9]. Enzymes currently on the market derive from mesophilic organisms. However, the enzymes isolated from extreme environments are endowed with unique catalytic properties, and might be of great interest in different sectors of biotechnology. 

In Bruno et al. 2019 [5], the authors described microbial enzymes with a potential biotechnological interest isolated in either Arctic or Antarctic environments. Polar marine environments are a promising research area for the discovery of enzymes with a potential industrial application. Cold-adapted enzymes are characterized by unique catalytic properties in comparison to their mesophilic homologues, i.e., higher catalytic efficiency, improved flexibility and lower thermal stability. These features make them particularly interesting for a potential commercial use in the food industry, agricultural production, synthetic biology, and biomedicine. Oxidoreductases, transferases, hydrolases, lyases, isomerases and ligases, and their proposed biotechnological applications, were reviewed in this paper. Hydrolases are the most abundant class of cold-adapted enzymes with a potential industrial application in the detergent, textile, paper, and food industries. For example, lactases or β-galactosidases, due to their capacity to reduce lactose intolerance, have attracted the interest of many research group and industries, and some of these isolated from the Antarctic marine *P. haloplanktis* LMG P-19143 were patented [10] and produced in large quantities by NutrilabNV (Bekkevoort, Belgium). Other enzymes have found application in molecular biology; a cold-adapted uracil-DNA glycosylase isolated from a psychrophilic Antarctic marine bacterium was released by New England Biolabs [11], a cold-active nuclease, isolated from an Antarctic marine psychrophile, *Shewanella* sp. strain Ac10, was developed by Takara-Clontech [12], and a recombinant alkaline phosphatase isolated from the Antarctic bacterial strain TAB5 was developed by New England Biolabs [13]. 

In Liu et al. 2019 [14], samples of seal and penguin feces, soil and marine sediment, collected from Fildes Peninsula in Antarctica were used as sources for the bioprospecting of chitinase-producing microorganisms. After optimizing the medium components and culture conditions, a cold-adapted strain belonging to *Pseudomonas* was identified as the best producer of chitinase, exhibiting more than 50% of its catalytic activity even at 0 °C. The crude chitinase showed the significant inhibition of fungi *Verticillium dahlia* CICC 2534 and *Fusarium oxysporum* f. sp. *cucumerinum* CICC 2532, which can cause cotton wilt and cucumber blight, respectively, suggesting that the cold strain may be a competitive candidate for biological control in agriculture, especially at low temperatures.

Jin et al. 2019 [15] discussed the potential industrial applications of extremozymes, as thermophilic, psychrophilic, halophilic and piezophilic enzymes. Deep-sea thermophiles, able to grow at high temperatures of 41–120 °C, produce thermostable proteolytic enzymes, attractive for use in the detergent, food and feed industries. For example, an α-amylase from the thermophile *Thermococcus* sp., isolated from a deep-sea hydrothermal vent, is on the market, and is named Fuelzyme^®^, released by Verenium Corporation (San Diego, CA, USA) [16]. Deep-sea psychrophiles, exhibiting an active metabolism even at −25 °C, are a promising source of industrial cold-active enzymes with applicability in the textile, detergent, beverage, food and biofuel industries. Deep-sea halophilic enzymes have particularly great potential in the production of biodiesel, polyunsaturated fatty acids and food, or in the treatment of waste water containing high salt concentrations and starch residues. Deep-sea piezophilic enzymes, living up to 70–110 MPa, have shown high efficiency in applications for food production, where high pressures are used for the processing and sterilization of food materials. 

Aguila-Torres and co-authors 2020 [17] reported a taxonomic identification and biochemical characterization of cypermethrin-degrading and biosurfactant-producing bacterial strains from samples collected in cypermethrin-contaminated marine sediment in Chilean northern Patagonia. This environment is considered extreme because of a wide range of temperatures, ranging from 4 to 20 °C, salinity, and a low nutrient availability. In addition, there has been an extensive use of cypermethrin as an antiparasitic pesticide in the salmon farming industry in northern Patagonia. Cypermethrin, used in agriculture and aquaculture, is considered a possible human carcinogen, able also to affect marine ecosystems. In northern Patagonia, a high concentration of cypermethrin has been reported in marine sediment. Among isolated strains, four strains exhibited the highest growth rate on cypermethrin, and high levels of biosurfactant production. An analysis of the genome sequence of these strains demonstrated the presence of genes encoding esterase, pyrethroid hydrolase and laccase, associated with the different biodegradation pathways of cypermethrin. 

Bioremediation has become an important tool for removing pollutants from contaminated environments, taking advantage of the presence of microorganisms and their ability to clean contaminated environments. Hydrocarbon-degrading bacteria are able to metabolize contaminants, developing specific pathways for sustaining their energetic and carbon requirements in the presence of them.

Varrella and colleagues 2020 [7] in their review described the environmental characteristics of DHABs, highlighting the unique bacterial lineages found in these extreme habitats. DHAB-derived microorganisms represent promising candidates for the bioremediation of oil hydrocarbons thanks to the presence of enzymes involved in pathways associated with hydrocarbon degradation. Proteobacteria represented by sulfate-reducing Deltaproteobacteria, as well as sulfur-oxidizing Gamma- and Epsilonproteobacteria, Actinobacteria, Deferribacteres and Euryarchaeota, were found across DHABs worldwide. In addition, viruses, found to be well-preserved in DHAB sediments, were able to control prokaryotic dynamics in these ecosystems. The variety of prokaryotes inhabiting DHABs represents an important source of polyextreme enzymes, such as esterase, lipase, α-amilase, pullulanase and xylanase, with applications in pharmaceutical, food, and beverage industrial processes, and many bioactive compounds with antiviral, antimicrobial and antitumor activities.

The marine chemical diversity varies from simple peptides and linear fatty acids to complex compounds such as terpenes, alkaloids and polyketides, etc., incorporating elements used in the marine environment as chemical defenses against predators. Natural products with different structures, able to perform a wide range of biological activities, make marine biomolecules valuable alternatives to many pathogenic bacteria and fungi, especially in the era of antimicrobial resistance. The most commonly reported activity is toward pathogenic agents, including viruses. Discovering novel and efficient antimicrobial molecules is becoming essential for natural-product chemistry, because of antibiotic-resistant microorganisms or even multidrug-resistance, and the development of new emerging infections. One example is represented by a potent antiviral agent isolated from the ascidian *Aplidium albicans*, which is under clinical trials in patients affected by Corona Virus SARS-CoV-2 [18]. Many papers in this issue are related to antimicrobial activity as well as anticancer activity. Cancer is the leading cause of death globally, and finding new molecules with anticancer activities remains a major challenge in the pursuit for a cure. 

Zain ul Arifeen et al. 2019 [19] wrote a review in which they described the structure, biological activity, and distribution of secondary metabolites produced by deep-sea fungi in the last five years. Fungi living in deep-sea environments produce unique secondary metabolites for defense and communication. Despite being the producer of many important bioactive molecules, deep-sea fungi have not been explored thoroughly due to methodological and technical limitations. However, their abundance and presence in all possible extreme ecosystems make them an ideal source of new bioactive molecules with many applications. Polyketide- and nitrogen-containing compounds, polypeptides, ester and phenolic derivatives, piperazine derivatives and terpenoid compounds were the bioactive molecules isolated from deep-sea fungi and showing antibiotic, antimicrobial, antiviral activities as well as cytotoxicity against cancer cells. Most of these compounds were isolated from two fungal genera, i.e., *Penicillium* and *Aspergillus*. Among these natural products, terpenoid derivatives were the most abundant compounds with the strongest antibiotic and cytotoxic activities with respect to other classes of molecules. In particular, breviones, isolated from the deepest sediment-derived fungus *Penicillium* sp. (5115 m depth), showing the strongest cytotoxic activity against cancer cells, have the potential to be good candidates for anticancer drugs. 

Corral and co-authors 2019 [20] described the antimicrobial and anticancer molecules produced by microorganisms, illustrating their action mechanisms *in vitro*. Halophilic microorganisms, such as archaea, bacteria and fungi, widely distributed around the world, inhabit hypersaline ecosystems characterized by a salinity higher than seawater, i.e., 3.5% NaCl. They are a source of bioactive molecules with applications in biomedicine. The continuous increase in antibiotic resistance establishes an urgent need for the exploiting of natural and sustainable resources to find novel antimicrobial molecules. Bacteria of the genus *Nocardiopsis* and *Streptomyces*, belonging to the phylum Actinobacteria, were found to be the main producers of antimicrobial compounds, whereas among fungi the genus *Aspergillus* was the most prolific. Likewise, *Nocardiopsis*, *Streptomyces*, *Bacillus*, *Halomonas* and *Aspergillus* were the most frequent producers of antitumoral molecules. Some of these compounds are promising candidates for preclinical trials.

Choi et al. 2019 [21] reported the characterization of deinoxanthin from a novel reddish *Deinococcus* sp. AJ005 isolated from seawater near King George Island in Antarctica, whose genome was recently completely sequenced. *Deinococcus* strains, Gram-positive bacteria, live in different habitats such as air, soils, and seas, and also at high altitudes and in Antarctic environments. They cope with these extreme habitats thanks to a variety of metabolic pathways, including the biosynthesis of antioxidants such as deinoxanthin. This carotenoid is particularly interesting for its use as an antioxidant, a cosmetic ingredient, and a food or feed additive, it being an efficient scavenger of reactive oxygen species and an anticancer agent. On the basis of genome annotation analysis, the authors proposed the deinoxanthin biosynthetic pathway, investigating the effects of culture conditions on the deinoxanthin biosynthesis in this strain.

John et al. 2020 [22] used a new *Pseudomonas* strain associated with the Antarctic marine ciliate *Euplotes focardii* to obtain silver nanoparticles (AgNPs) after incubation with 1 mM of AgNO_3_ within 24 h. Nanoparticles (NPs) have become particularly interesting in biomedical sciences, drug-gene delivery, space industries, cosmetics and chemical industries. AgNPs show general antibacterial and bactericidal features, thus becoming promising tools in biomedical applications. Since the physiochemical methods used for AgNP synthesis are not convenient given their high energy consumption and the use of toxic reagents, there has been a growing need to develop a simple and low-cost approach to AgNP synthesis without toxic chemicals. An alternative to the chemical synthesis method is to use microbes to obtain nanoparticles. This easy and efficient biological method to synthesize AgNPs may be used against drug-resistant pathogenic bacteria, contributing to solving the problem of antibiotic resistance. The authors characterized the size and morphology of AgNPs and demonstrated that *Pseudomonas* AgNPs showed a higher antibacterial activity against *Escherichia coli*, *Staphylococcus aureus* and *Candida albicans* with respect to the chemically synthesized NPs. The results of this paper are related to the patent number 102019000014121 deposited in 06/08/2019. 

Only two articles in this Special Issue are focused on macroorganisms, specifically the deep-sea Antarctic sponge *Latrunculia biformis*, collected from the Antarctic Weddell Sea shelf at a depth of 291 m, and the Antarctic krill *Euphausia superba* that represents the most abundant biomass in cold environments at the base of the Antarctic food chain.

In Li et al. 2019 [23], the authors isolated diverse discorhabdin alkaloids from the Antarctic deep-sea sponge *L. biformis*. Three known discorhabdins, (−)-discorhabdin L, (+)-discorhabdin A and (+)-discorhabdin Q, and three new discorhabdin analogs (−)-2-bromo-discorhabdin D, (−)-1-acetyl-discorhabdin L and (+)-1-octacosatrienoyl-discorhabdin L, were identified and characterized by bioactivity and molecular networking-based metabolomics and the chemical structures elucidated by extensive spectroscopy analyses. (−)-discorhabdin L, (−)-1-acetyl-discorhabdin L and (+)-1-octacosatrienoyl-discorhabdin L showed promising anticancer activities, demonstrated by the molecular modeling of the potential binding of discorhabdins to the anticancer targets involved in their anticancer activity. (−)-1-acetyl-discorhabdin L and (+)-1-octacosatrienoyl-discorhabdin L are the first discorhabdin analogs with an ester function at C-1 with (+)-1-octacosatrienoyl-discorhabdin L, which is the first discorhabdin bearing a long-chain fatty acid at this position.

The experimental article by Huang et al. 2020 [24] reported the first genome survey of the Antarctic krill *E. superba*, a very important marine organism in the Antarctic food chain. The high-throughput comparative identification of putative antimicrobial peptides (AMPs) and antihypertensive peptides (AHTPs) from whole-body transcriptomes of the Antarctic krill and its mesophilic counterpart, the whiteleg shrimp *Penaeus vannamei*, revealed that AMPs/AMP precursors and AHTPs were generally conserved, with interesting variations between the two crustacean species due to cold adaptation. This paper is a preliminary exploration of bioactive peptides in a polar key species of the trophic chain for the development of novel marine drugs.

## 3. Perspectives and Conclusions

The papers included in this Special Issue provide an overview of the growing interest in species biodiversity, highlighting the importance of marine extreme environments as sources of a unique marine chemical diversity of molecules. It is worth noting that six articles in this Issue are focused on molecules and enzymes isolated from Antarctica. This means that there is an increasing interest in this habitat because it is perceived as an important source of drug discovery. In fact, the unique environment and ecological pressures of marine polar regions might be the major drivers of a selection of unique biological communities able to biosynthesize new compounds with diverse biological activities. It is expected that, in the near future, more marine molecules from polar regions as well as from other extreme habitats will find their way into biomedical and biotechnological applications.

In conclusion, the Guest Editor thanks all the authors that contributed with their interesting articles to this Special Issue, all the reviewers for evaluating the submitted manuscripts, and the Editorial board of Marine Drugs, the Editor-in-Chief of the Journal Orazio Taglialatela-Scafati for their support, especially in this difficult period of the 2020 pandemic SARS CoV-2. 
